# Nuclease activity and protein A release of *Staphylococcus aureus* clinical isolates determine the virulence in a murine model of acute lung infection

**DOI:** 10.3389/fimmu.2023.1259004

**Published:** 2023-10-02

**Authors:** Nadine Ludwig, Julia Thörner-van Almsick, Sina Mersmann, Bernadette Bardel, Silke Niemann, Achmet Imam Chasan, Michael Schäfers, Andreas Margraf, Jan Rossaint, Barbara C. Kahl, Alexander Zarbock, Helena Block

**Affiliations:** ^1^Department of Anesthesiology, Intensive Care and Pain Medicine, University Hospital Muenster, Muenster, Germany; ^2^Institute for Medical Microbiology, University Hospital Muenster, Muenster, Germany; ^3^Institute of Immunology, University Hospital Muenster, Muenster, Germany; ^4^European Institute for Molecular Imaging (EIMI), University of Muenster, Muenster, Germany

**Keywords:** neutrophil recruitment, lung infection, Staphylococcus aureus, neutrophil extracellular traps, SpA, L-selectin shedding, TNFR shedding

## Abstract

*Staphylococcus aureus* is a common cause of hospital-acquired pneumonia associated with high mortality. Adequate clinical treatment is impeded by increasing occurrence of antibiotic resistances. Understanding the underlying mechanisms of its virulence during infections is a prerequisite to finding alternative treatments. Here, we demonstrated that an increased nuclease activity of a *S. aureus* isolate from a person with cystic fibrosis confers a growth advantage in a model of acute lung infection compared to the isogenic strain with low nuclease activity. Comparing these CF-isolates with a common MRSA-USA300 strain with similarly high nuclease activity but significantly elevated levels of Staphylococcal Protein A (SpA) revealed that infection with USA300 resulted in a significantly increased bacterial burden in a model of murine lung infection. Replenishment with the cell wall-bound SpA of *S. aureus*, which can also be secreted into the environment and binds to tumor necrosis factor receptor -1 (TNFR-1) to the CF-isolates abrogated these differences. *In vitro* experiments confirmed significant differences in *spa*-expression between USA300 compared to CF-isolates, thereby influencing TNFR-1 shedding, L-selectin shedding, and production of reactive oxygen species through activation of ADAM17.

## 1 Introduction

*Staphylococcus aureus* is a Gram-positive bacterium that may cause a variety of infections, including skin and soft tissue infections, sepsis, and pneumonia. *S. aureus* is a common cause of hospital-acquired infections and is associated with an increased morbidity and mortality. Dissemination of *S. aureus* may result in sepsis with a high mortality rate of up to 30% (1, 2). Especially the development of antibiotic resistances is a major health care problem. *S. aureus* USA300, first described in 2000 in the USA, was initially resistant to methicillin, but further resistances emerged over the years. Throughout the years it was distributed globally and was associated with skin and soft tissue infection, but also invasive diseases such as bacteremia, endocarditis, or necrotizing pneumonia (3, 4).

*S. aureus* is also one of the most commonly isolated bacteria of people suffering from cystic fibrosis (pwCF). CF is a genetic disorder caused by mutations of the CF transmembrane conductance regulator gene on chromosome 7, affecting the production and secretion of mucus in the lungs, pancreas, and other organs (5). The thick and sticky mucus in the lungs provides an ideal environment for some CF-related bacteria, including *S. aureus*, which may colonize and infect the airways (6). Its presence is associated with increased inflammation, rapidly deteriorating lung functionality, and worsened clinical outcome (7, 8). *S. aureus* is known to carry a plethora of virulence factors, toxins, and mechanisms contributing to evasion of host response mechanisms and potentially increasing its virulence (9). The most prominent example is the small β-barrel pore-forming toxin α-hemolysin, which not only lyses red blood cells, but also impacts a wide range of nucleated cells, and has been shown to contribute to skin necrosis and lethal infections (10). Another factor receiving more and more attention is staphylococcal protein A (SpA), which belongs to the microbial surface components recognizing adhesive matrix molecules (MSCRMMs) (11). SpA binds non-specifically to the Fc-regions of IgGs, thereby hindering phagocytosis (12). Additionally, it suppresses the host immune response by early shedding of the tumor necrosis factor receptor -1 (TNFR-1) from the cell surface in an ADAM17-dependent manner, thus, preventing downstream signaling (13) and neutralizing soluble TNFα in the inflammatory environment (14). Furthermore, the expression of nucleases by *S. aureus* has been suggested to serve as an evasion mechanism, since elimination of nucleases in *S. aureus* resulted in decreased bacterial burden during pneumonia (15), or peritonitis (16). Nucleases can degrade the DNA backbone of Neutrophil Extracellular Traps (NETs) which are formed in response to invading pathogens. In contact to *S. aureus* neutrophils release decondensed chromatin fibers in a NADPH oxidase-dependent manner decorated with histones and granule proteins, such as myeloperoxidase, neutrophil elastase, and cathelicidins (17, 18), facilitating the immobilization and elimination *in vivo* and *in vitro* (19, 20). A recent study analyzed *S. aureus* isolates from sputum, deep throat, and nasal swab samples formerly collected from one pwCF during a period of 15 years (21). The persisting *S. aureus* strains developed an increased nuclease expression over the years, indicating that nuclease activity supports the evasion from the host’s immune response, and facilitate long term survival of the pathogen within the patient. *In vitro* experiments indicated that isolates with a high nuclease activity confer a survival advantage in the presence of NETs compared to isolates with low nuclease activity (21). This study was designed to analyze the role of nucleases for acute lung infection in more detail using *S. aureus* isolates that express nucleases with different activities. Thus, we compared an early isolate of 2001 with low nuclease activity with a later isolate of 2012 exhibiting high nuclease activity (21). Whole genome sequencing revealed clonality of these isolates, characterizing them as ideal candidates to investigate the role of nucleases at physiological levels without any genetic manipulation (21). Additionally, the two clinical isolates belong to multi locus sequence type (MLST) ST30 and spa type t617 (21). ST30 is a common MLST type throughout the world. Many *S. aureus* strains of this ST (22) belong to the group of community-acquired methicillin-resistant *S. aureus* (caMRSA), which is common in the US and in other parts of the world indicating that the data shown here are relevant for many *S. aureus* strains. We analyzed the impact of the different nuclease activities on neutrophil recruitment, and bacterial burden in a murine model of acute lung infection. We compared the results of the CF isolates with results of the strain USA300, which is a common methicillin-resistant strain in the US and well established for the induction of pneumonia in mice (15, 23). This strain occurs worldwide (24) in the context of healthcare-acquired infections, and is associated with a complicated treatment due to antibiotic resistances (25). Our results revealed a significant worse outcome for the USA300 infected mice compared to the clinical isolates. Significant differences in SpA release influencing TNFR-1, L-selectin shedding and production of reactive oxygen species (ROS) were detected between USA300 and the clinical isolates, indicating an impact on its virulence. Understanding the mechanisms of *S. aureus* pathogenesis and developing effective therapies to combat *S. aureus* infections is critical for improving patients’ outcomes.

## 2 Materials and methods

### 2.1 *S. aureus* strains and growth conditions

*S. aureus* strain AH1263 (USA300 CA-MRSA ErmS) (3) and isolates from a pwCF with the numbers 17 (isolated in 2001) and 81 (isolated in 2012) with same spa-type t617 but different nuclease activities (21) were cultivated on Columbia 5% blood agar plates (Thermo scientific), in the following referred to as low_17_, and high_81_, respectively. Strains were grown in brain heart glucose bouillon (BHI, Roth) at 37°C at 160 rpm. To determine the number of colony forming units (CFUs) of *S. aureus*, serial dilutions were plated on Luria-Bertani Agar plates and incubated over night at 37°C. For direct use of *S. aureus* in co-culture with murine bone marrow derived neutrophils (BMDNs), bacteria were counted using a Neubauer improved counting chamber.

### 2.2 Nuclease assay and SpA ELISA

To assess the differential nuclease activity of the *S. aureus-* isolates, 2.5 µl of sterile filtered *S. aureus* supernatants were incubated with 7µg calf thymus DNA for 1h at 37°C in a total volume of 50 µl 300 mM Tris-HCl supplemented with CaCl_2_ and MgCl_2_ (each 3 mM) and subjected to agarose gel electrophoresis (15). Densitometry analysis was performed using ImageJ. To analyze the amount of SpA in the supernatant of *S. aureus* USA300, low_17_ and high_81_ were cultivated for 24 h, the supernatant collected, sterile filtered, and 5 µl were used for a Protein A ELISA (abcam) according to the manufacturer’s instructions.

### 2.3 Mice

We used 8–16 week old C57BL/6 mice, both male and female with a weight range between 18 g and 28 g. The mice were kept in a barrier facility under specific pathogen-free (SPF) conditions. All animal experiments were approved by the LANUV NRW.

### 2.4 Preparation of BMDNs

Femur and tibia of C57Bl/6 mice were flushed with 6 ml PBS. This cell solution was carefully resuspended and layered on top of 6 ml 62% Percoll in PBS with 20 mM HEPES and centrifuged at 2500 rpm, for 30 min at room temperature without brake. The supernatant was removed and the neutrophil containing layer at the bottom of the tube was washed with PBS. Cells were counted and adjusted as described in the following method sections.

### 2.5 Co-incubation of murine neutrophils with *S. aureus* or SpA

To analyze the capability of BMDNs to form NETs and inhibit growth of *S. aureus in vitro*, Percoll-purified BMDNs (2x10^6^/ml in RPMI1640, 1% FCS, 10 mM HEPES, 1 mM CaCl_2_, 1 mM MgCl_2_) were co-incubated with *S. aureus* low_17_ and high_81_ for 1 or 2 h with a multiplicity of infection (MOI) of 10 at 37°C with gentle shaking (50 rpm). At these time points, cultures were gently detached from the well surface, and an aliquot was used to determine the CFUs by plating of serial dilutions. Here, the cell suspension was thoroughly mixed by vortexing and pipetting to ensure optimal dispersion of bacteria. In some experiments, cells and bacteria were co-incubated for 90 min and half of the samples were treated with DNaseI (Sigma, 120 U/ml) and incubated for further 45 min at 37°C. For L-selectin shedding analysis, BMDNs were incubated with purified SpA (Sigma, P3838, 1 µg/1x10^5^ BMDNs) or H_2_O as vehicle control (2.5% v/v) for 10 min or 30 min at RT. Following centrifugation (400 g, 5 min), BMDNs were used for flow cytometry or ImageStream^®X^ imaging flow cytometry analysis to assess or visualize NET formation (anti-Ly6G, clone 1A8, BioLegend; anti-MPO clone 2D4, abcam; Sytox red, ThermoFisher), or L-selectin surface expression (anti-CD62L, clone DREG56, BioLegend), and the supernatant was used for the NET-specific ELISA.

### 2.6 NET-specific ELISA

To analyze the abundance of NET-fragments in solutions, an ELISA was performed directed against the NET-specific citrullinated histone H3. For capturing, a 96-well plate (Immunolon 4HBX) was coated overnight at 4°C with 50 µl/well of H3cit-antibody (4 µg/ml, ab5103, abcam) in coating buffer (15 mM Na_2_CO_3_, 35 mM NaHCO_3_, pH 9.6), washed 3x with PBS, and blocked with 5% BSA/PBS for 2 h. Wells were washed 3x (1% BSA/PBS, 0.02% Tween20), and 50 µl of the respective samples were applied to each well and incubated for 2 h. After an additional washing step, 50 µl of the detection antibody (anti-DNA-POD, Cell death Detection ELISA Plus, Roche, 1:100 dilution in 1% BSA/PBS) was applied to the wells and incubated for 2 h, shaking at 140 rpms at room temperature. After 5 washes, 100 µl/well TMB-substrate (ThermoFisher) were added, incubated for 20 min at RT in the dark. Absorbance at 652 nm was measured using the Synergy Mx microplate reader.

### 2.7 Lung infection with *S. aureus*

Overnight cultures of *S. aureus* were diluted to an OD of 0.1, incubated for further 3.5 h, and diluted to an OD of 1 in 10 ml BHI medium. Bacteria were pelleted, washed with 10 ml ice cold PBS and finally resuspended in 10 ml PBS, and stored at 4°C overnight. Serial dilutions were plated to assess the number of CFUs in these cultures. C57BL/6 mice were anesthetized by intraperitoneal injection of ketamine (125 µg/g body weight, Pfizer), and xylazine (12.5 µg/g body weight, Bayer). The trachea was exposed, and 50 µl of the *S. aureus* suspension (6x10^8^ CFUs/mouse in PBS) or vehicle control were administered via a 29-gauge needle. In some experiments, 6x10^8^ CFUs were resuspended in PBS containing 50 µg purified SpA (13) (Sigma, P-3838). After 4 h or 24 h respectively, mice were sacrificed, blood was collected, and the lungs lavaged 4 times with 0.7 ml saline. Following lung perfusion with 3 ml PBS, lungs were enzymatically digested with hyaluronidase type I-s, collagenase type XI, and DNase I (Sigma), and filtered through a nylon mesh (70 µm) to achieve single cell suspensions. The number of neutrophils in the bronchoalveolar lavage (BAL) fluid and the lung was determined by using a Sysmex hemacytometer, and further discriminated by flow cytometry using an anti-CD45, an anti-Gr-1, and an anti-Ly6B.2 antibody. CFUs within the BAL, the lung, and the blood were assessed by serial plating. Total protein content within the BAL was determined by using a BCA-assay (Pierce). TNFR- or L-selectin shedding in the BAL or in the plasma was assessed using a DuoSet ELISA (DY425, DY576 R&D) according to the manufacturer’s instructions.

### 2.8 Release of reactive oxygen species

The release of superoxide in response to *S. aureus* was measured by a cytochrome c reduction assay. Percoll-purified BMDNs were resuspended in HBSS supplemented with 20 mM HEPES at a density of 3.95 x10^6^/ml and pre-incubated at 37°C for 60 min with SpA (Sigma; 1 mg/ml in H_2_O; 1 µg/1x10^5^ cells), H_2_O (7.2% v/v) or DMSO (0.5% v/v) as vehicle controls, or the matrixmetalloproteinase inhibitor GM6001 (abcam, 50 µM final concentration). Afterwards, 270 µl of BMDNs were well mixed with 30 µl *S. aureus* with an MOI of 10 and seeded in fibrinogen-precoated (3 h, 0.15 mg/ml HBSS) Immunolon-4HBX 96-well plates (70 µl/well). Cofactors such as CaCl_2_ (1 mM), MgCl_2_ (1 mM) and cytochrome c (0.1 mM) were added with or without TNFα (0.5 µg/ml) or superoxide dismutase (SOD, Sigma, 45 units). Absorbance was analyzed in a microplate reader (Mx Synergy) at 37°C at 550 nm and 490 nm every 4 min for a total time of 90 min. For calculation of nmoles O_2_^-^ each measurement was corrected by its SOD control measurement and subjected to the Beer-Lambert law.

### 2.9 Statistics

Results are presented as means ± standard error mean (s.e.m.). Differences between groups were analyzed using Student´s t-test, one-way ANOVA, or one-way ANOVA with Dunnett´s multiple comparisons, where appropriate using GraphPad Prism Software v9.3. A p-value of ≤ 0.05 was considered statistically significant.

## 3 Results

### 3.1 Increased nuclease activity confers a growth advantage *in vitro* and *in vivo*

**Figure 1:**
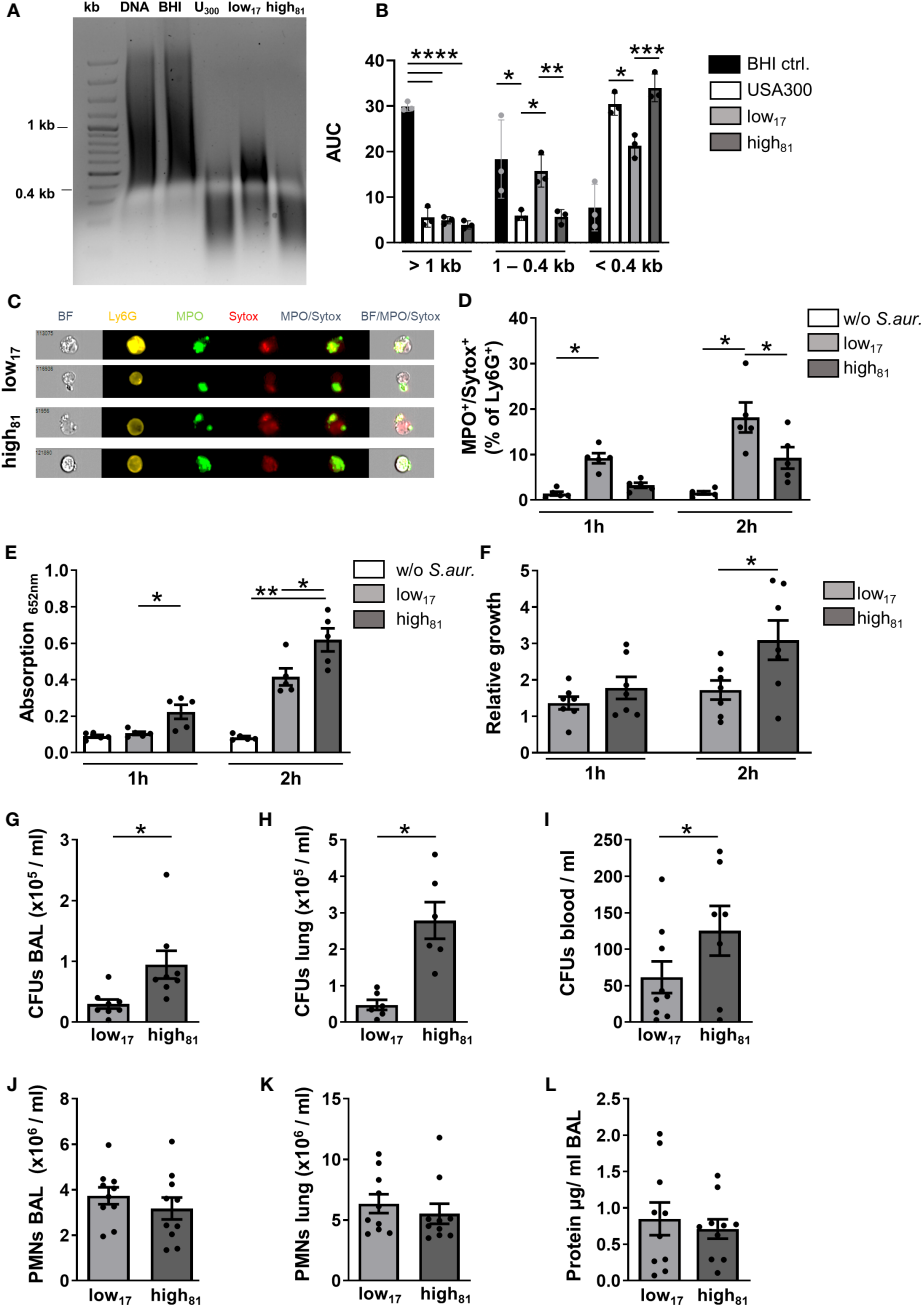
Increased nuclease activity confers a growth advantage *in vitro* and *in vivo*. **(A)** Representative agarose gel showing calf thymus DNA samples after incubation with the supernatant of *S. aureus* cultures USA300, low_17_ or high_81_ or BHI medium as control. **(B)** Densitometric analysis of agarose gels with digested calf thymus DNA following incubation with *S. aureus* supernatants. n=3. **(C)** Representative ImageStream^®X^ images of MPO^+^/Sytox^+^ BMDNs following incubation with *S. aureus* low_17_ and high_81_. BMDNs were co-cultivated with *S. aureus* CF-isolates low_17_ or high_81_ at an MOI of 10. **(D)** Ly6G^+^ neutrophils were analyzed by flow cytometry for the percentage of cells stained positive for MPO and Sytox as a readout for NET release. **(E)** The supernatant of these co-cultures was used for a NET-specific ELISA (capturing: H3-cit, abcam; detection: anti-DNA, Roche). **(F)** Number of CFUs at the respective time points divided by the number of CFUs seeded at the beginning of the co-incubation are presented as relative growth after 1 h or 2 h of co-cultivation. Data are mean +/- sem; n=5-7; *p < 0.05, **p < 0.01, Two-way ANOVA. **(G–L)** Analysis of CFU and neutrophil recruitment 24 h after intratracheal instillation of 6x10^8^ bacteria. Serial dilutions of BAL **(G)**, lung homogenate **(H)** or blood **(I)** were plated to assess the number of CFUs. Flow cytometry analysis revealed the number of neutrophils recruited to the alveoli **(J)** or the lung tissue **(K)**. The protein content in the BAL was analyzed using a BCA protein assay **(L)**. Data are presented as mean +/- sem; n=8-10; *p < 0.05, ***p < 0.001, ****p < 0.0001; One-way ANOVA or student's t-test.

To confirm the increased nuclease activity of high_81_ compared to low_17_ as described before (21), calf thymus DNA was incubated with the supernatant of *S. aureus* cultures or BHI medium as a control, respectively. To estimate the activity, the digested DNA was subjected to gel electrophoresis and analyzed for the abundance of DNA fragments at different sizes. The representative image shown in **Figure 1A** and the densitometry analysis (**Figure 1B**) clearly demonstrate the significantly lower amount of small DNA fragments following incubation with the supernatant of the low_17_ isolate, compared to a common USA300 strain or the high_81_ isolate (**Figure 1B**).

To determine the capability of murine neutrophils to produce NETs in response to *S. aureus*, we co-cultivated BMDNs together with the two different CF isolates, and analyzed them via flow cytometry for a positive staining of myeloperoxidase (MPO), a typical component of NETs, and Sytox, which detects extracellular DNA. ImageStream^®X^ analysis revealed that extracellular fibers were stained positive for both, indicating that *S. aureus* stimulation of BMDNs induced NET formation (representative image shown in **Figure 1C**). No significant differences regarding the morphology, staining intensity, or the area of positive signals were observed following incubation with low_17_ or high_81_ (data not shown). Following 1 h of co-incubation with *S. aureus* low_17_, flow cytometry analysis revealed a significant increase of MPO^+^/Sytox^+^ neutrophils compared to unstimulated control cells, with a further rise after 2 h of incubation (**Figure 1D**). Following incubation with high_81_, a significant increase compared to control cells was detected after 2 h, whereas this increment was significantly lower compared to neutrophils incubated with low_17_ (**Figure 1D**). Interestingly, incubation of BMDNs with the USA300 strain resulted in significantly more MPO^+^/Sytox^+^ neutrophils compared to the high_81_ isolate, although they exhibited similar nuclease activities (**Supplementary Figure S1A**). In contrast to the flow cytometry, which focusses on the analysis of MPO^+^/Sytox^+^ cells still retaining their morphology, the NET specific ELISA detects NET-fragments in the supernatant of the co-cultures. Here, we observed significantly more NET fragments following incubation with *S. aureus* high_81_, compared to control cells as well as to *S. aureus* low_17_ stimulated neutrophils (**Figure 1E**), indicating that higher nuclease activities shed the NETs from the neutrophils, leading to higher amounts of NET fragments in the supernatant. To investigate whether the different nuclease activities influence the neutrophil-mediated killing or growth inhibition of *S. aureus*, we compared the CFUs following co-incubation of BMDNs with these two *S. aureus* isolates. Here, we observed a significant increase of CFUs of the high_81_ -isolate after 2 h, in contrast to low_17_, with only a modest increase of CFUs compared to the amount of seeded CFUs in this co-culture (**Figure 1F**). Incubation with USA300 resulted in a similar relative growth as observed for the low_17_ isolate (**Supplementary Figure S1B**), indicating that strain specific attributes influence growth in this setting. These results suggest that the higher nuclease activity of the high_81_ isolate diminishes the ability of neutrophils to kill or inhibit growth of *S. aureus*. To further confirm that the increased amounts of CFUs following incubation with high_81_ was not due to an increased nuclease-mediated bacterial dispersion, we added DNaseI to these samples and observed no significant differences compared to the CFUs of non-treated samples (**Supplementary Figure S1C**).

Since this increase of nuclease activity was suggested to be an adaptive response to the neutrophil-rich and inflammatory environment within pwCF, we wanted to analyze the influence of this increased nuclease activity in a model of acute lung infection in mice. The blood was collected, the lung was lavaged, harvested and analyzed for the number of CFUs 24 h after intratracheal instillation with each of these two *S. aureus* isolates (**Figures 1G–I**). Interestingly, we observed a significant increase in the number of CFUs for the high_81_ strain in BAL (**Figure 1G**), lung (**Figure 1H**), and blood (**Figure 1I**). Analyzing the number of neutrophils in the BAL (**Figure 1J**) or the lung tissue (**Figure 1K**) revealed that the two isolates do not display any differences in their ability to induce neutrophil recruitment. Intratracheal instillation of PBS as vehicle control causes recruitment of only minor numbers of neutrophils (**Supplementary Figure S1D**). Furthermore, although we observed increased CFUs for the high_81_ isolate-infected mice, the protein content in the alveolar space was not affected (**Figure 1L**). However, although high amounts of bacteria were injected, mice dealt surprisingly well with the induced lung infection and none of the mice died. These observations led us to the question, how these isolates differ from the common MRSA USA300 strain.

### 3.2 Mice infected with *S. aureus* USA300 develop significantly worsened pneumonia compared to mice infected with CF-isolates low_17_ and high_81_

**Figure 2:**
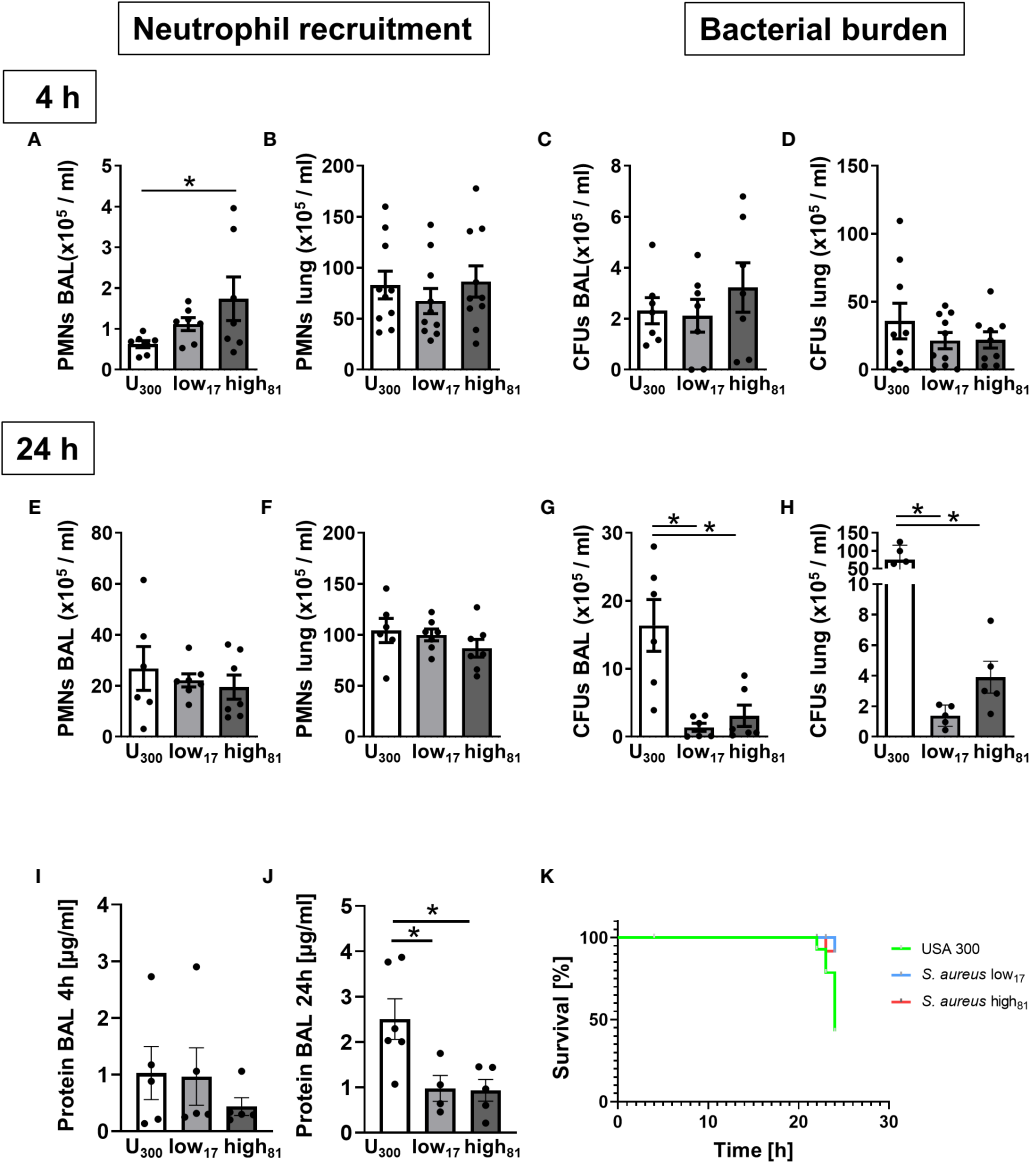
Mice infected with USA300 develop significantly worsened pneumonia compared to mice infected with CF-isolates low_17_ and high_81._ Analysis of neutrophil recruitment and CFUs 4h **(A–D)** or 24 h **(E–H)** after intratracheal instillation of *S. aureus* (6x10^8^/mouse). Neutrophil recruitment to the alveoli **(A, E)** or the lung **(B, F)** and bacterial burden in the alveoli **(C, G)** or the lung **(D, H)** was determined by flow cytometry and plating serial dilutions. The protein content in the BAL was assessed using a BCA assay **(I, J)**. Number of moribund or dead mice are illustrated by a Kaplan-Meier Survival curve **(K)**. Data are presented as mean +/- sem; n = 6-10; *p < 0.05 One-way ANOVA.

To assess the virulence of the CF-isolates low_17_ and high_81_, both strains were compared to the USA300 *S. aureus* strain in a model of acute lung infection. To investigate the early phase following infection, mice were analyzed after 4 h regarding neutrophil recruitment to the alveoli (**Figure 2A**) and the lung (**Figure 2B**). Here, we observed less recruited neutrophils in the BAL of USA300-infected mice compared to the CF-isolates, but no differences within the lung tissue (**Figure 2B**). However, the amount of CFUs in the BAL (**Figure 2C**) and the lung (**Figure 2D**) was similar in all mice at this time point. Extending the observation interval to 24 h revealed that infection with USA300 did not induce an altered recruitment of neutrophils to the alveoli (**Figure 2E**), or the lung (**Figure 2F**). Interestingly, evaluation of the CFU numbers in the BAL (**Figure 2G**) or the lung (**Figure 2H**) revealed a significant increase in USA300 infected mice. Assessing the protein content in the alveolar space after 4 h, no differences were observed (**Figure 2I**). In contrast, the protein content in the BAL collected from USA300 infected mice 24 h after infection, displayed a significant increase (**Figure 2J**). In line with these observations, we surveyed significantly more animals with a severe burden, which had to be euthanized for ethical reasons, in those groups infected with USA300 (**Figure 2K**). These data suggest a reduced virulence of the CF-isolates compared to the USA300 strain.

### 3.3 Release of SpA correlates with the amount of sL-sel and sTNFR-1

**Figure 3:**
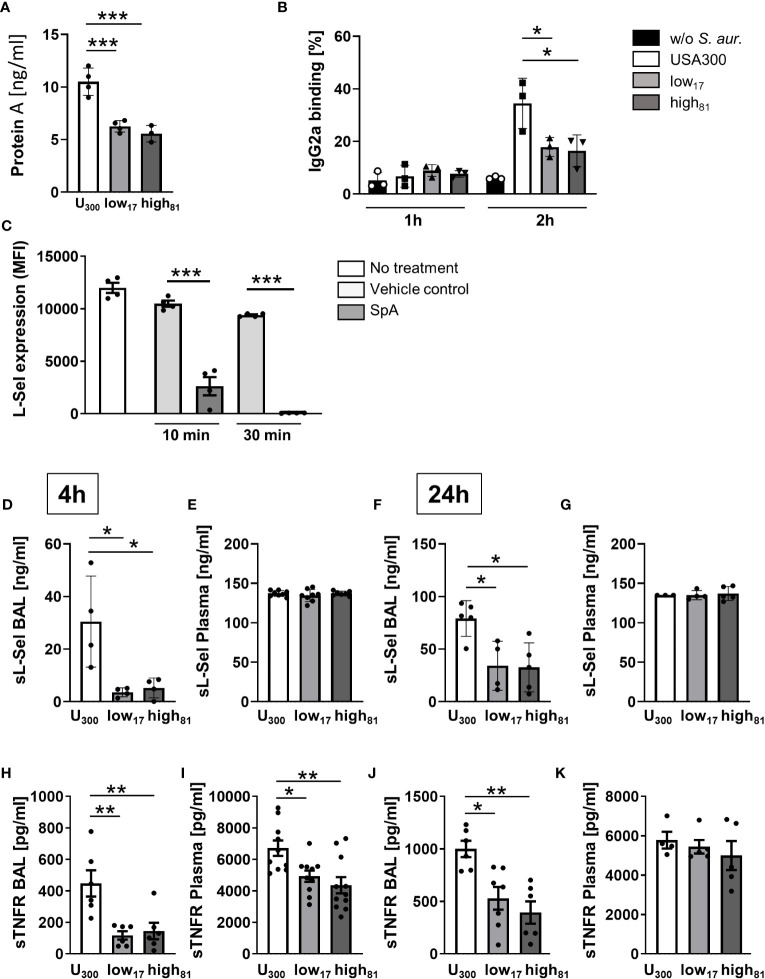
Release of protein A correlates with the amount of sL-sel and sTNFR. **(A)** Amount of Protein A in the supernatant of *S. aureus* cultures after 24 h of cultivation. **(B)** Flow cytometric analysis of bound IgG2a antibody binding to Ly6G^+^ neutrophils. **(C)** Flow cytometry analysis of L-Selectin expression on Ly6G^+^ neutrophils left untreated or after incubation with water as vehicle control or 1 µg purified SpA for the indicated timepoints. **(D–G)** sL-sel ELISA of BAL and plasma samples of *S. aureus* infected mice 4h **(D, E)** or 24h **(F, G)** after infection. **(H–K)** sTNFR ELISA of BAL and plasma samples of *S. aureus* infected mice 4 h **(H, I)** or 24 h **(J, K)** after infection. Data are presented as mean +/- sem; n = 6-10; *p < 0.05, **p < 0.01, ***p < 0.001 One-way ANOVA.

*S. aureus* is known for its large variety of proteins contributing to its toxicity and virulence. One of these factors is SpA, which has been demonstrated to induce TNFR-1 shedding, at least partially through ADAM17 (13, 14). This metalloproteinase also influences L-selectin shedding, contributing to the host response during lung infection as demonstrated recently (26). To further elucidate the role of SpA in the immune response against invading *S. aureus*, we first analyzed the secretion of SpA of the USA300 strain in comparison to the two CF-isolates and observed significantly more SpA release by the USA300 strain compared to both isolates (**Figure 3A**). Accordingly, investigating the percentage of IgG2a-positive neutrophils, indicative of SpA binding or interaction, in the co-culture with these three *S. aureus* strains revealed a significant increase of IgG2a-positive cells for the USA300 strain compared to control cells or neutrophils incubated with the CF-isolates low_17_ and high_81_ after 2 h (**Figure 3B**). Incubation of BMDNs with recombinant SpA induced fast shedding of L-selectin from the neutrophil surface (**Figure 3C**), indicating ADAM17 activity. To confirm the physiological relevance *in vivo*, we analyzed the BAL and the plasma samples from mice infected with the three *S. aureus* strains for the abundance of soluble L-Selectin (sL-Sel) using an ELISA assay (**Figures 3D–G**). Indeed, mice infected with the USA300 strain displayed significantly more sL-Sel in the BAL (**Figure 3D**), but not in the plasma (**Figure 3E**) 4 h post infection. Similarly, analyzing the amount of sL-Sel 24 h post infection revealed significantly more sL-Sel in the BAL (**Figure 3F**), but not in the plasma (**Figure 3G**) compared to those mice which were infected with low_17_ or high_81_. No differences were observed between these two CF-isolates. To further corroborate these findings, we also analyzed the amount of soluble TNFR (sTNFR) in these samples. Interestingly, the abundance of sTNFR in USA300 infected mice in comparison to mice infected with low_17_ and high_81_ was significantly increased after 4 h in the BAL (**Figure 3H**) and plasma (**Figure 3I**). However, analyzing the infected mice after 24 h revealed a less pronounced, but still significant difference between USA300 and low or high-infected mice in the BAL (**Figure 3J**), whereas no significant difference was observable in the plasma (**Figure 3K**).

### 3.4 TNFα-primed BMDNs challenged with USA300 produce significantly more ROS compared to BMDNs challenged with CF-isolates

**Figure 4:**
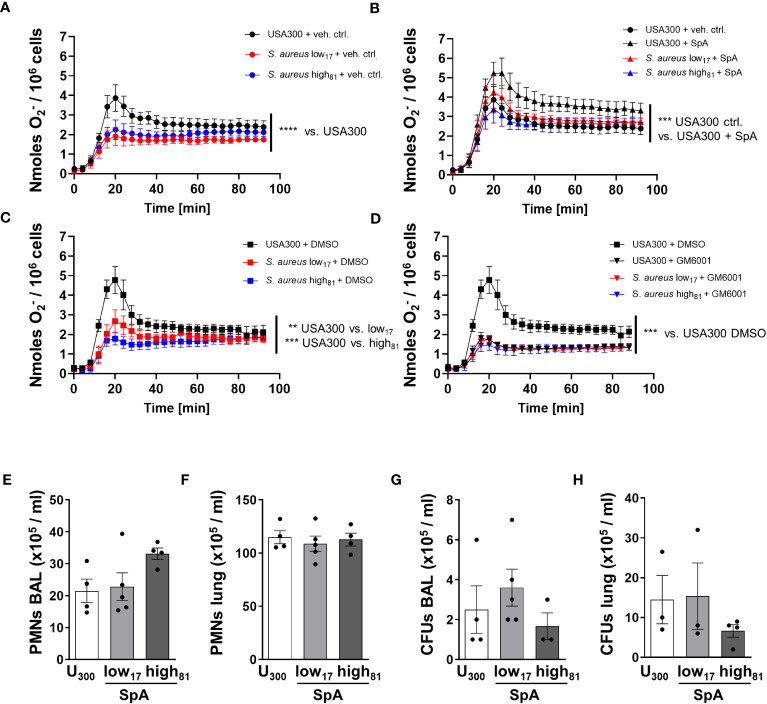
TNFα-primed BMDNs challenged with USA300 produce significantly more ROS compared to BMDNs challenged with CF-isolates. O_2_^-^ release of BMDNs in response to TNFα, fibrinogen, and *S. aureus.* BMDNs were pre-incubated with **(A)** vehicle control H_2_O (7.2% v/v), **(B)** SpA (1 µg/1x10^5^ cells), **(C)** vehicle control DMSO (0.5%), or **(D)** ADAM17 inhibitor GM6001 (50 µM); n=4. **(E–H)** Analysis of neutrophil recruitment and bacterial burden 24 h after intratracheal instillation of 6x10^8^ USA300 or *S. aureus* low_17_ and high_81_ supplemented with 50 µg SpA per mouse. Neutrophil recruitment to the alveoli **(E)**, to the lung **(F)** and bacterial burden within the BAL **(G)** or the lung **(H)** were analyzed. Data are presented as mean +/- sem; n = 3-5; *p < 0.05, **p < 0.01, ***p < 0.001 One-way ANOVA; Dunnett’s Multiple Comparison Test (for **A–D**).

Co-stimulation of TNFα-primed BMDNs with fibrinogen and USA300 and H_2_O (7.2% v/v) as vehicle control induced the release of ROS, whereas neutrophils challenged with the two CF-isolates had a significantly weaker response (**Figure 4A**). To assess whether the formation of ROS in this assay is influenced by the amount of SpA, the same assay was performed with neutrophils pre-incubated with recombinant SpA (**Figure 4B**). Here, we observed a significant increase of ROS formation compared to control cells without additional SpA (**Figure 4A**). The response of neutrophils to both isolates, low_17_ and high_81_, is similar compared to neutrophils incubated with USA300 without additional SpA, while the addition of SpA to BMDNs in the presence of USA300 further increased the production of ROS. In order to confirm that the difference between USA300 and low_17_ or high_81_ induced ROS formation is dependent on the activation of ADAM17, BMDNs were pre-incubated with the vehicle control DMSO, which did not further influence ROS production (**Figure 4C**), or the matrix metallopeptidase inhibitor GM6001, which inhibits also ADAM17 activity (**Figure 4D**). Here, we observed that inhibition of ADAM17 in USA300 stimulated neutrophils reduced ROS production to the levels of those neutrophils challenged with the CF-isolates. Interestingly, no strain-specific or SpA-/GM6001- mediated differences were visible in BMDNs without TNFα priming (**Supplementary Figures S2A, B**). To exclude that an altered uptake of bacteria might influence ROS production, we performed a phagocytic assay. BMDNs were incubated with the *S. aureus* isolates and analyzed for the number of intracellular bacteria following different times of incubation, revealing no significant differences (**Supplementary Figure S2C**).

We further investigated whether the addition of SpA during infection of mice might influence the disease, and worsen the outcome of those mice being infected with CF-isolates low_17_ and high_81_. WT mice were either infected with USA300 or one of the clinical CF-isolates low_17_ and high_81_ supplemented with 50 µg SpA. 24 h following infection mice were analyzed for neutrophil recruitment to the alveoli (**Figure 4E**) or the lung (**Figure 4F**) and their bacterial burden (**Figures 4G, H**). Similar to previous experiments, we did not observe any significant differences regarding neutrophil recruitment to the alveoli (**Figure 4E**) or the lung (**Figure 4F**), although there was a tendency towards an increased neutrophil recruitment in the BAL in response to high_81_ following supplementation with SpA. However, analyzing the bacterial burden of the BAL (**Figure 4G**) and the lung (**Figure 4H**) revealed no significant differences between the USA300 infected mice and those mice which received one of the isolates supplemented with SpA, indicating that SpA abolishes the previously observed difference in virulence between USA300 compared to low_17_ or high_81_. Interestingly, the addition of SpA also eliminates the growth advantage of the high_81_ compared to the low_17_ isolate, which might also be attributable to the slightly higher number of recruited neutrophils.

## 4 Discussion

Understanding the molecular mechanisms of *S. aureus* pathogenesis is a prerequisite for the development of novel therapies for the prevention and treatment of acute and chronic *S. aureus* infections. The use of *S. aureus* isolates from a pwCF, which developed an increased nuclease activity over the years, enabled us to investigate the effect of a physiologically adapted nuclease activity on their survival in the presence of murine neutrophils *in vitro* and *in vivo*. Previous studies already demonstrated a survival advantage of high nuclease isolates in the presence of NETs generated by stimulation of human neutrophils with PMA (21). In the current study, we induced NET formation in murine BMDNs by co-culture with *S. aureus* CF-isolates, and analyzed their survival capacity within this co-culture. In concordance with the previously published data in human neutrophils, the high_81_ isolate displayed a significant growth advantage compared to the low_17_ isolate. Determination of NETs sticking to the surface of neutrophils in comparison to NET fragments in the supernatant supports the hypothesis that increased nuclease activity leads to an accelerated shedding of NETs from the neutrophil surface resulting in smaller fragments.

Investigating the effect of the increased nuclease activity in a murine model of acute lung infection also revealed a growth advantage of *S. aureus* high_81_. This is in line with two other studies, analyzing mutant *S. aureus* strains lacking nuclease activity in acute lung infection (15), and in a model of murine peritonitis (16). These studies demonstrated that nuc-deficient *S. aureus* (USA300-AH1680) was killed more effectively by the host compared to the respective WT strain (USA300-AH1263). Our study is the first using clonal identical *S. aureus* clinical isolates with physiological variations of nuclease activity without any genetic modifications, thereby confirming the previously published data relying on genetically modified *S. aureus* strains. However, although *in vitro* controls indicate that addition of DNaseI to the samples did not influence CFU analysis, it cannot be excluded that an increased entanglement of *S. aureus* low_17_ to longer NET fibers within the alveoli influenced the CFU numbers in the BAL.

Interestingly, in our model of acute lung infection, the nuclease activity alone does not influence the survival of those mice infected with each of the clinical isolates, which might be due to a non-functional important virulence regulator *agr* already in the early isolate (27, 28). The down-regulation of the *agr* locus during chronic airway infection in CF has been observed before (29). While the *agr* regulator has an important function for the expression of various virulence genes, inactivity of the *agr* locus by either down-regulation or by mutations facilitate persistence of the bacteria in tissues. Goerke et al. also showed that the activity of *agr* was independent of the expression of α-toxin, which is positively regulated by *agr* and which has an important function for the pathogenesis of *S. aureus*-related pneumonia (30), and spa transcription, which is suppressed by *agr*, in sputa of pwCF (29).

Comparing these isolates with a USA300 strain of *S. aureus* revealed obvious differences during pneumonia. USA300 infection was associated with a massively increased bacterial burden, significant increase of protein content in the BAL, and a significantly worsened outcome within 24 h. Interestingly, assessment of neutrophil recruitment into the alveoli 4 h after infection showed a decreased cellular infiltration following USA300 infection in contrast to those mice infected with one of the CF-isolates, probably enabling an early bacterial proliferation. However, recruitment to the lung tissue after 4 h, or into the alveoli or the lung after 24 h was not different between all strains. Virulence of *S. aureus* has been associated with many different factors, toxins, and defense mechanisms, such as SpA (9). Indeed, the SpA content of the supernatants of USA300 cultures was significantly increased compared to the cultures of clinical isolates. Accordingly, significantly more BMDNs displayed attached SpA following co-incubation of neutrophils with the USA300 strain compared to the CF-isolates, indicating that SpA is able to bind to neutrophils. SpA can impact host immune defense against *S. aureus* in various ways. On the one hand neutrophils and keratinocytes respond to SpA by secreting pro-inflammatory cytokines such as IL8, TNFα, or MIP1α *in vitro*, and the release of NETs (31, 32). On the other hand SpA is known to protect *S. aureus* from IgG-mediated phagocytosis, to induce shedding of the TNFR, and was suggested to neutralize TNFα (12–14). Surprisingly, we did not observe differences regarding phagocytosis between USA300 and low_17_ or high_81_, most likely due to differences within the experimental setting. SpA induces TNFR shedding through ADAM17 activation (33). Previous studies demonstrated, that ADAM17 also regulates L-selectin shedding (26). Indeed, we were able to demonstrate that purified SpA incubation resulted in fast L-selectin shedding from the neutrophil surface *in vitro*. Accordingly, the amount of soluble L-selectin in the BAL of USA300 infected mice was significantly higher compared to those mice infected with the CF-isolates low_17_ or high_81_. Similar results were obtained analyzing the amount of soluble TNFR. Here, in BAL and plasma sTNFR levels were significantly higher in USA300 infected mice. Interestingly, for both molecules the difference between USA300- and low_17_/high_81_-infected mice declines between 4 h and 24 h, indicating that the SpA mediated ADAM17 activation influences rather the early phase of disease. This is in line with studies investigating ADAM17 null mutant mice in a model of *Escherichia coli* induced peritonitis (34). Here, loss of ADAM17 led to an early increase of neutrophil recruitment to the peritoneal cavity 2 h after infection, whereas it turned into a decreased recruitment compared to WT mice after 24 h. At both time points, ADAM17-deficient mice displayed a significantly reduced bacterial burden. In contrast, Cappenberg et al. demonstrated a reduced neutrophil recruitment in ADAM17 deficient mice to the lung following infection with *Klebsiella pneumonia* after 24 h, associated with increased bacterial burden and severely affected neutrophil effector functions, attributed to the loss of L-selectin shedding through ADAM17 (26). However, another study described that incubation with gram-negative bacterial strains such as *Pseudomonas aeruginosa* or *E. coli* did not induce early shedding of TNFR (14), indicating that strain- and organ-specific differences might also play an important role. Our studies revealed that co-incubation of BMDNs with USA300 *in vitro* led to significantly more ROS production than incubation with each of the CF-isolates. Supplementation of the CF-isolates with purified SpA led to increased ROS production at similar levels as generated following incubation with USA300 without additional SpA; and *vice versa*, inhibition of ADAM17 in neutrophils incubated with USA300 reduced the ROS response to the levels of those neutrophils responding to the CF-isolates, supporting the hypothesis that SpA influences ROS generation through ADAM17 mediated L-selectin shedding. These data are the first describing a direct correlation between SpA, ADAM17, and ROS production. Interestingly, this effect was only observed in TNFα-primed neutrophils, raising the question whether SpA might induce a different signaling cascade in the presence of excessive amounts of TNFα. The mechanism behind this effect should be addressed in future studies.

However, although higher amounts of ROS were generated in response to USA300 *in vitro*, this effector function could not contribute to an efficient removal of bacteria *in vivo*. It is likely that the infected mice would have required the recruitment of more neutrophils at the early phase of disease to combat the excessive growth of USA300, what might have been hindered by activation of ADAM17 via SpA. Additionally, the requirement of TNFα for the SpA induced elevated ROS response might equally come into play; it was immediately available in high concentrations *in vitro*, whereas *in vivo* it might be limited in the early phase of disease, especially if immune cell recruitment is delayed. Furthermore, sTNFR can neutralize TNFα, as demonstrated before in a murine model of systemic infection with *S. aureus* and a respective *spa*-deficient mutant, lacking SpA expression (14). In our studies, replenishment of SpA during the infection of mice with low_17_ and high_81_ bacteria worsened the outcome of these mice leading to a similar bacterial burden as those mice infected with USA300, indicating that SpA is a dominant factor of the altered virulence. However, it cannot be excluded that other virulence factors such as toxins differ between these strains and contribute to the observed phenotype. Surprisingly, the higher nuclease activity did not play any role in this setting, which might be due to the higher number of recruited neutrophils in response to high_81_ infection, or the physiological increase of nuclease activity might be only relevant up to a certain threshold of bacterial load. Another explanation could be the overall less virulence of the early isolate with low nuclease activity due to the lack of presence and/or expression of the well characterized virulence factors for acute pneumonia in addition to SpA such as α-toxin (Hla) and Panton-Valentin Leucocidin (PVL) (30, 35, 36).

Taken together, our data raise the question, why the *S. aureus* strain with a lower virulence in the mouse model persists for years in a highly inflammatory milieu present in pwCF. On the one hand it might be that the murine immune system acts different from the human immune system on *S. aureus* infiltration, especially on isolates adapted to humans. On the other hand, it is possible that the compromised immune system of pwCF is not capable to eradicate *S. aureus* completely and that its low virulence facilitates the survival of such strains by staying unrecognized.

## Data availability statement

The raw data supporting the conclusions of this article will be made available by the authors, without undue reservation.

## Ethics statement

The animal study was approved by Landesamt für Natur, Umwelt und Verbraucherschutz NRW. The study was conducted in accordance with the local legislation and institutional requirements.

## Author contributions

NL: Investigation, Writing – review & editing. JTvA: Investigation, Writing – review & editing. SM: Investigation, Writing – review & editing. BB: Investigation, Writing – review & editing. SN: Writing – review & editing. AC: Investigation, Writing – review & editing. MS: Conceptualization, Writing – review & editing. AM: Writing – review & editing, Investigation. JR: Conceptualization, Writing – review & editing. BK: Conceptualization, Writing – review & editing. AZ: Conceptualization, Funding acquisition, Supervision, Writing – review & editing. HB: Conceptualization, Funding acquisition, Investigation, Supervision, Writing – original draft, Writing – review & editing.

## Supplementary material

Supplementary Figure 1 NET formation, relative growth of *S. aureus* and PMN recruitment during lung infection. **(A)** Ly6G^+^ neutrophils were analyzed by flow cytometry for the percentage of cells stained positive for MPO and Sytox as a readout for NET release following incubation with *S. aureus* USA300, low_17_ or high_81_ for the indicated time points. **(B)** Number of CFUs at the respective time points divided by the number of CFUs seeded at the beginning of the co-incubation are presented as relative growth after 1 h or 2 h of co-cultivation as mean +/- sem, n=4, Two-way ANOVA *p < 0.05, **p < 0.01, ***p < 0.001, ****p < 0.0001. **(C)** Number of CFUs following co-incubation of BMDNs with *S. aureus* USA300, low_17_ or high_81_ for 135 min with a multiplicity of infection (MOI) of 10. Within the last 45 min half of the samples were incubated with 120 units/ml DNaseI (Sigma). Data are presented as mean +/- sem; n = 4. No significant differences were detectable. **(D, E)** Analysis of neutrophil recruitment to the alveoli **(D)** or the lung tissue **(E)** 24 h after intratracheal instillation of PBS as vehicle control or 6x10^8^ bacteria.

Supplementary Figure 2 BMDNs show no differences in ROS production or phagocytosis in response to *S. aureus* incubation. **(A, B)** O_2_^-^ release of BMDNs in response to fibrinogen, and *S. aureus* without TNFα priming. BMDNs were pre-incubated with vehicle control H_2_O, or SpA **(A)** for 1 h at 37°C, or with the vehicle control DMSO or ADAM17 inhibitor GM6001 **(B)** for 1 h at 37°C; Data are presented as mean +/- sem; n=4. **(C)** Number of CFUs of previously internalized bacteria following co-incubation of BMDNs with *S. aureus* (MOI = 10). Lysostaphin treatment (20 µg/ml) eliminated non-internalized bacteria and H_2_O incubation lysed BMDNs for release of bacteria. Data are presented as mean +/- sem; n = 6. Two-way ANOVA. No significant differences were detectable.
