# Supplementary material for: Nuclease activity and protein A release of Staphylococcus aureus clinical isolates determine the virulence in a murine model of acute lung infection
Source: Front Immunol. 2023 Oct 2;14:1259004. doi: 10.3389/fimmu.2023.1259004 (PMC10577289; doi:10.3389/fimmu.2023.1259004)
Supplement: Supplementary file 1 [file DataSheet_1.docx]

**Supplemental methods**

**Phagocytosis**

For opsonisation, *S. aureus* was incubated for 30 min at 37°C in RPMI supplemented with 10% murine serum. Following Percoll purification, 5x10^5^ BMDNs in RPMI + 10% FCS were incubated with the respective *S. aureus* strains for the indicated time points with an MOI of 10. Following incubation, 20 µg/ml Lysostaphin was added to the samples and incubated for 15 min at room temperature to kill non-internalized bacteria. Cells were centrifuged and resuspended in 1ml ice-cold H_2_O for 15 min at 4°C to lyse BMDNs and release internalized bacteria. Afterwards, serial dilutions were plated to assess CFUs, indicating the amount of phagocytosed bacteria. Relative growth was determined by dividing the CFUs after incubation with the CFUs before incubation.
