# Supplementary figures and images for: Nuclease activity and protein A release of Staphylococcus aureus clinical isolates determine the virulence in a murine model of acute lung infection
Source: Front Immunol. 2023 Oct 2;14:1259004. doi: 10.3389/fimmu.2023.1259004 (PMC10577289; doi:10.3389/fimmu.2023.1259004)

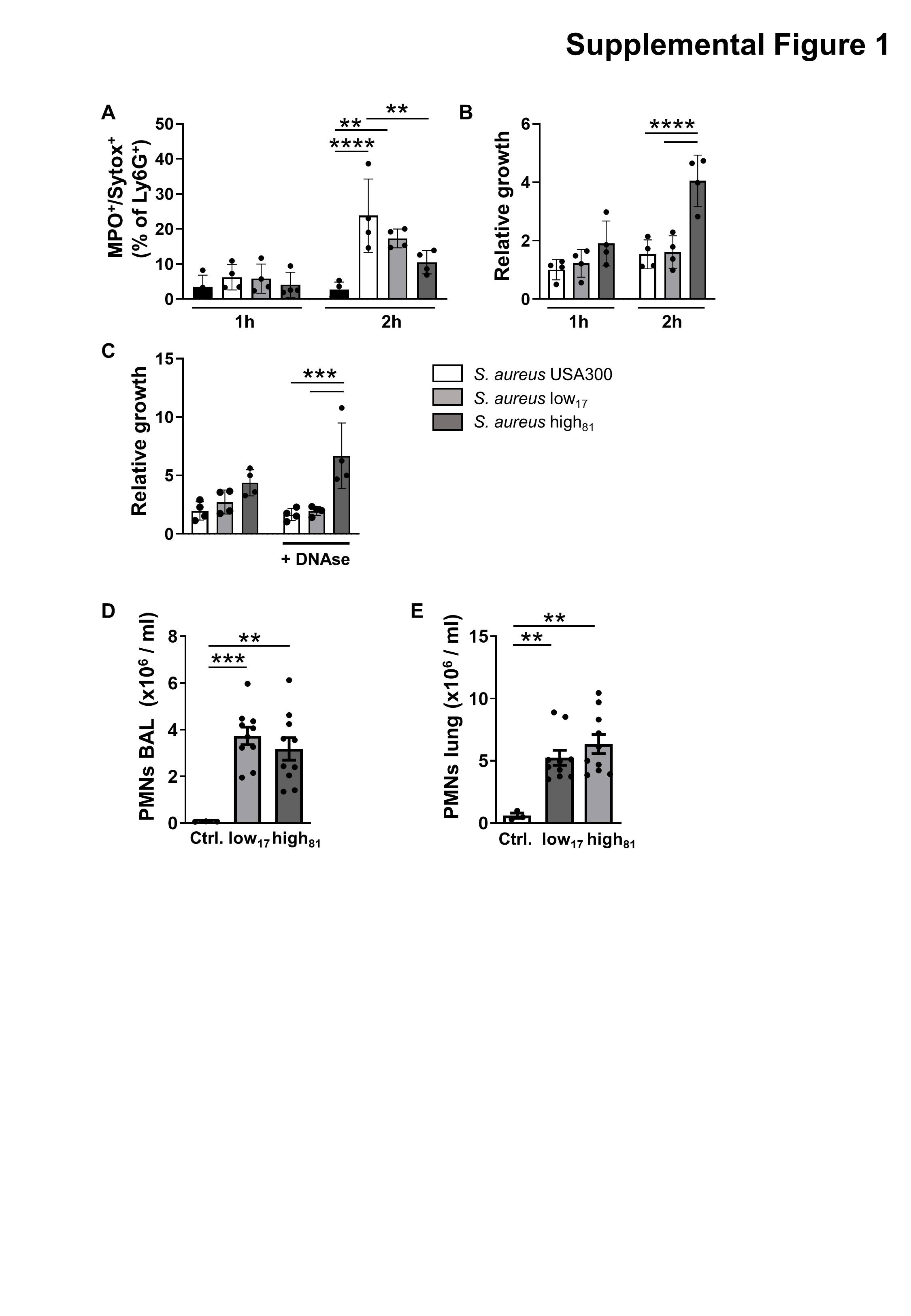

Supplement: Supplementary Figure 1 — NET formation, relative growth of S. aureus and PMN recruitment during lung infection. (A) Ly6G+ neutrophils were analyzed by flow cytometry for the percentage of cells stained positive for MPO and Sytox as a readout for NET release following incubation with S. aureus USA300, low17 or high81 for the indicated time points. (B) Number of CFUs at the respective time points divided by the number of CFUs seeded at the beginning of the co-incubation are presented as relative growth after 1 h or 2 h of co-cultivation as mean +/- sem, n=4, Two-way ANOVA *p < 0.05, **p < 0.01, ***p < 0.001, ****p < 0.0001. (C) Number of CFUs following co-incubation of BMDNs with S. aureus USA300, low17 or high81 for 135 min with a multiplicity of infection (MOI) of 10. Within the last 45 min half of the samples were incubated with 120 units/ml DNaseI (Sigma). Data are presented as mean +/- sem; n = 4. No significant differences were detectable. (D, E) Analysis of neutrophil recruitment to the alveoli (D) or the lung tissue (E) 24 h after intratracheal instillation of PBS as vehicle control or 6x10^8^ bacteria. [file Image_1.tif]

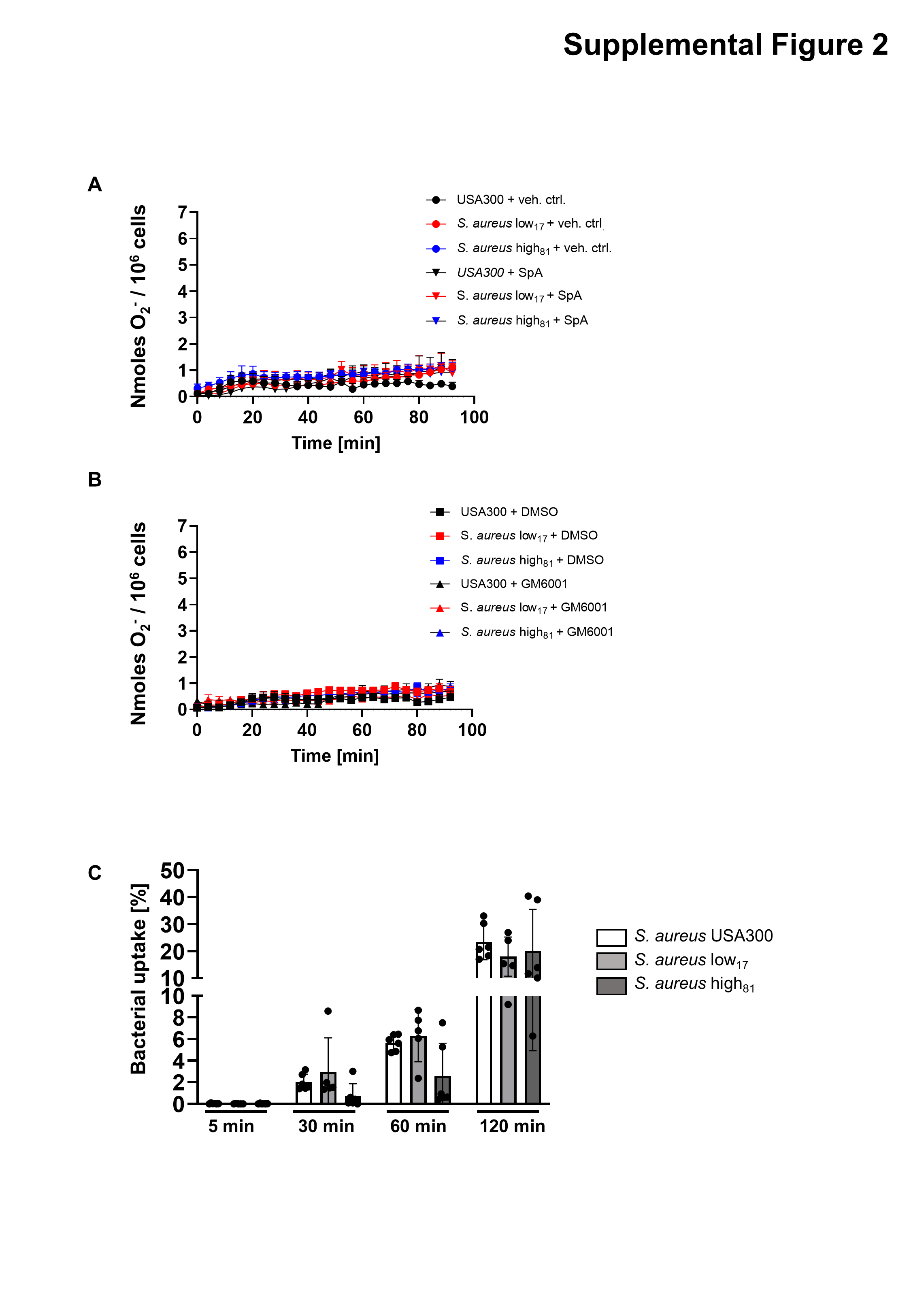

Supplement: Supplementary Figure 2 — BMDNs show no differences in ROS production or phagocytosis in response to S. aureus incubation. (A, B) O2- release of BMDNs in response to fibrinogen, and S. aureus without TNFα priming. BMDNs were pre-incubated with vehicle control H2O, or SpA (A) for 1 h at 37°C, or with the vehicle control DMSO or ADAM17 inhibitor GM6001 (B) for 1 h at 37°C; Data are presented as mean +/- sem; n=4. (C) Number of CFUs of previously internalized bacteria following co-incubation of BMDNs with S. aureus (MOI = 10). Lysostaphin treatment (20 µg/ml) eliminated non-internalized bacteria and H2O incubation lysed BMDNs for release of bacteria. Data are presented as mean +/- sem; n = 6. Two-way ANOVA. No significant differences were detectable. [file Image_2.tif]
